# Transcription Factors Oct-1 and GATA-3 Cooperatively Regulate Th2 Cytokine Gene Expression via the RHS5 within the Th2 Locus Control Region

**DOI:** 10.1371/journal.pone.0148576

**Published:** 2016-02-03

**Authors:** Kiwan Kim, Najung Kim, Gap Ryol Lee

**Affiliations:** Department of Life Science, Sogang University, Seoul, Korea; Jawaharlal Nehru University, INDIA

## Abstract

The T helper type 2 (Th2) locus control region (LCR) regulates Th2 cell differentiation. Several transcription factors bind to the LCR to modulate the expression of Th2 cytokine genes, but the molecular mechanisms behind Th2 cytokine gene regulation are incompletely understood. Here, we used database analysis and an oligonucleotide competition/electrophoretic mobility shift assays to search for transcription factors binding to RHS5, a DNase I hypersensitive site (DHS) within the Th2 LCR. Consequently, we demonstrated that GATA-binding protein-3 (GATA-3), E26 transformation-specific protein 1 (Ets-1), octamer transcription factor-1 (Oct-1), and Oct-2 selectively associate with RHS5. Furthermore, chromatin immunoprecipitation and luciferase reporter assays showed that Oct-1 and Oct-2 bound within the *Il4* promoter region and the Th2 LCR, and that Oct-1 and GATA-3 or Oct-2 synergistically triggered the transactivational activity of the *Il4* promoter through RHS5. These results suggest that Oct-1 and GATA-3/Oct-2 direct Th2 cytokine gene expression in a cooperative manner.

## Introduction

CD4 T cells are important for mediating adaptive immune responses against various pathogens [1]. Naïve CD4 T cells differentiate into several subsets, including T helper type Th (1), Th2, Th17, and regulatory T cells under the influence of cytokines during T cell receptor (TCR) activation [2]. Transcription factors drive various differentiation programs by inducing subset-specific gene expression during cell differentiation. Each subset of cells secretes specific sets of cytokines and has its own function in immune responses [2].

Th2 cells secrete interleukin (IL)-4, IL-5, and IL-13, and are involved in humoral immunity and allergic responses. The cytokine genes, *Il4*, *Il13*, and *Il5*, are located in close proximity to each other within a small region, termed the Th2 cytokine locus, in human chromosome 5 and mouse chromosome 11. These genes are expressed in Th2 cells, but are silenced in other cell types. Epigenetic modifications, such as DNase I hypersensitivity, DNA demethylation, restriction enzyme accessibility, histone acetylation, and histone methylation all contribute to the Th2 cell differentiation [3]. The Th2 cytokine gene locus is controlled by several regulatory elements, encompassing transcriptional enhancers, transcriptional silencers, and the locus control region (LCR). Among them, the Th2 LCR directs the coordinate expression of Th2 cytokine genes through epigenetic changes [4].

The Th2 LCR is located within the 3’ region of the *Rad50* gene and includes four DNase I hypersensitivity sites (DHSs), RHS4, RHS5, RHS6, and RHS7 [3,5]. We previously showed that Th2 cell differentiation is defective in CD4-specific Th2 LCR-deficient mice, demonstrating that the Th2 LCR is important in the regulation of Th2 cytokine genes [6]. Some of the DHSs in the Th2 LCR have been investigated by analysis of transgenic mice or regulatory region-knockout mice. For example, RHS7 is rapidly demethylated in Th2 cells and exhibits strong enhancer activity in transgenic mice [7]. However, deletion of RHS7 shows only a partial effect on IL-4 and IL-13 and no effect on IL-5 expression under Th2-polarizing conditions [5]. On the other hand, deletion of RHS6 more potently inhibited the gene expression of all three Th2 cytokine genes [8]. However, the role of RHS5 in the modulation of Th2 cytokine genes has not been thoroughly investigated.

Several transcription factors are essential for the regulation of the Th2 LCR. We previously revealed that GATA-binding protein-3 (GATA-3), a so-called “master regulator” of Th2 cells, binds to several regulatory regions within the Th2 cytokine locus, including the Th2 LCR. Moreover, these transactivational activities are antagonized by metastasis-associated protein-2 (MTA-2) binding within the same region [9]. Ying Yang 1 (YY1) is also required for the regulation of Th2 cytokine expression, and exerts its actions via binding to the Th2 LCR and cooperation with GATA-3 [10].

The purpose of this study was to uncover molecular mechanisms fundamental to the control of Th2 cytokine gene expression. To this end, we employed database analysis and an electrophoretic mobility shift assay to search for transcription factors binding to RHS5. We also employed chromatin immunoprecipitation and luciferase reporter assays to reveal that Oct-1 binds to RHS5 and other sites within the Th2 cytokine gene locus, and that Oct-1 cooperates with GATA-3 or Oct-2 to direct Th2 cytokine synthesis.

## Materials and Methods

### Mice

All animal experiments were performed in accordance with the Sogang University (Seoul, Korea) Institutional Animal Care and Use Committee (IACUC) guidelines. Experiments with live mice were approved by the Institutional Animal Care and Use Committee of Sogang University (Permit number: SGU2010-03). Six-week-old C57BL/6 mice were purchased from Orient Bio (Republic of Korea) and maintained in the Sogang University animal facility under specific-pathogen free conditions. The mice were sacrificed by CO_2_ inhalation when necessary.

### Cell culture

The T lymphocyte EL4 cell line and the Phoenix-Eco cell line were purchased from the Korean Cell Line Bank (Laboratory of Cell Biology, Cancer Research Center and Cancer Research Institute, Seoul National University College of Medicine, Seoul, Korea). The EL4 cell line was maintained in Dulbecco’s modified Eagle’s medium (DMEM) supplemented with 10% horse serum (Invitrogen, Carlsbad, CA, USA) and 1% penicillin/streptomycin (Invitrogen), and the Phoenix-Eco cell line was maintained in DMEM supplemented with 10% fetal bovine serum (Invitrogen) and 1% penicillin/streptomycin.

### Transcription binding site analysis

Analysis of putative transcription factor binding sites *in silico* in RHS5 was performed using Alibaba2.1 software (http://www.gene-regulation.com/pub/programs.html#alibaba2)

### *In vitro* CD4 T cell differentiation

Naïve CD4 T cells were stimulated and differentiated *in vitro* as previously described [9]. Briefly, splenocytes were obtained from 6-week-old C57BL/6 mice, and red blood cells were removed by treatment with ACK (ammonium-chloride-potassium) lysis buffer (Invitrogen). Monoclonal antibodies directed against major histocompatibility complex class II (107610, BioLegend), natural killer cell 1.1 antigen (108712, BioLegend), cluster of differentiation (CD) 8 (100735, BioLegend) and CD25(102031, BioLegend) were incubated with the cells for 30 min on ice for negative selection, followed by depletion of the negatively selected cells with a mixture of magnetic beads conjugated to anti-rat and anti-mouse Ig antibodies (Perseptive Biosystems, Framingham, MA, USA). Biotin-conjugated anti-CD62L antibody (104404, BioLegend) and magnetic bead-conjugated anti-biotin antibody (130-090-485, Miltenyi Biotec) were then used to sort naïve CD4 T cells. The naïve cells were stimulated with plate-bound anti-CD3 antibody (10 μg/ml) (100302, BioLegend), soluble anti-CD28 antibody (2 μg/ml) (102102, BioLegend) and 20 U/ml of IL-2 (14-8021-64, eBioscience) in RPMI medium in 10% fetal bovine serum (Invitrogen) and penicillin/streptomycin. For Th1 cell polarization, IL-12 (3.5 ng/ml) (14-8121-80, eBioscience) and anti-IL-4 antibody (504102, BioLegend) were added to the RPMI medium under the same culture conditions, and for Th2 cell polarization, IL-4 (1,000 U/ml)(14-8021-64, eBioscience) and anti-IFN-γ antibody (505802, BioLegend) were added to the medium.

### Preparation of nuclear extracts and electrophoresis mobility shift assay (EMSA)

For the preparation of the nuclear extracts, *in vitro*-differentiated Th1 or Th2 cells were resuspended in buffer A (10 mM HEPES, pH 7.9, 10 mM KCl, 0.1 mM dithiothreitol (DTT), and 0.5 mM phenylmethylsulphonyl fluoride (PMSF)), gently mixed by vortexing, and kept on ice for 15 min. Next, 10% Nonidet^™^ P-40 (NP-40) (Sigma-Aldrich, St. Louis. MO, USA) was added to the cell lysate and strongly mixed by vortexing for 10 sec. Pellets were obtained by centrifugation, extracted by vortexing in buffer B (20 mM HEPES, pH 7.9, 0.4 M NaCl, 1 mM ethylene diamine tetraacetic acid (EDTA), 1 mM DTT, 1 mM PMSF, and 10% NP-40) for 10 min, and centrifuged. The resulting supernatants were used as the nuclear extracts.

The binding of transcription factors to their response elements was measured by EMSA. Nuclear extracts (2 μg) were incubated with 1 μg of poly dI:dC (Sigma-Aldrich) and ^32^P-labeled oligomers for 30 min in binding buffer (10 mM Tris-HCl, pH 7.5, 50 mM NaCl, 1 mM DTT, 1 mM EDTA, and 5% glycerol) on ice. For the antibody-supershift assay, 5 μg of anti-Oct-1 (sc232X, Santa Cruz Biotechnology, Santa Cruz, CA, USA), anti-Oct-2 (sc233X, Santa Cruz Biotechnology), anti-GATA-3 (sc9009X, Santa Cruz Biotechnology), or anti- E26 transformation-specific protein 1 (Ets-1) (sc55581X, Santa Cruz Biotechnology) antibody was added to the mixture. The mixtures were resolved by sodium dodecyl sulfate (SDS)-polyacrylamide gel electrophoresis and subjected to autoradiography. The oligomers employed for EMSA are listed in S1 Table.

### Chromatin immunoprecipitation assay

Th1 and Th2 cells (1 × 10^6^) were cross-linked with 1% formaldehyde and quenched with 0.125 M glycine. Cells were lysed with a buffer containing 1% SDS and sonicated for 15 min by using a Bioruptor sonicator (Diagenode, Inc., Denville, NJ, USA). Cell extracts were pre-cleared with protein A/G agarose for 1 h and incubated with anti-Oct-1 antibody (sc232X, Santa Cruz Biotechnology), anti-Oct-2 antibody (sc233X, Santa Cruz Biotechnology), or negative control rabbit IgG (sc-2027, Santa Cruz Biotechnology) for immunoprecipitation overnight. Antibody-bound chromatin was precipitated with protein A/G agarose and eluted with sodium bicarbonate-SDS. The chromatin was reverse cross-linked by incubating at 65°C overnight. The amount of precipitated DNA was quantified by real-time polymerase chain reaction (PCR) using the SYBR green method with the primers listed in S2 Table.

### Retroviral transduction

The expression vectors for Oct-1 and Oct-2 were constructed from a MIEG3-based retroviral construct expressing enhanced green fluorescent protein (GFP). Phoenix-Eco cells (1 × 10^6^) were plated onto a 60 mm cell culture dish. After overnight culture, each retroviral vector (Oct-1-MIEG3, Oct-2-MIEG3, or empty MIEG3) together with the pCL-Eco helper vector were transfected into the cells. After 24 h, the culture supernatant was exchanged with fresh culture medium, and the new culture supernatant containing retrovirus was collected 24 h later. Purified naïve CD4 T cells were activated with plate-bound anti-CD3 antibody (5 μg/ml), anti-CD28 antibody (1 μg/ml), and murine IL-2 (1 μg/ml) for 24 h. Next, the retrovirus in the culture supernatant was transduced into activated CD4 T cells by spin-infection with polybrene (4 μg/ml) at 1,500 × g for 90 min at 32°C.

### Intracellular cytokine staining

Naïve CD4 T cells were activated, transduced as above, and differentiated into Th1- or Th2-polarizing conditions for 3 days. The Th1 and Th2 cells were then re-stimulated for 4 h with 1 μM ionomycin (Sigma-Aldrich) and 10 nM phorbol-12-myristate-13-acetate (PMA) (Sigma-Aldrich) with Golgi stop solution (BD, Franklin Lakes, NJ, USA). For intracellular cytokine staining, Th1/Th2 cells were fixed/permeabilized by using a BD intracellular cytokine staining kit for 20 min. The fixed cells were stained with anti-IL-4-antibody-phycoerythrin conjugate (anti-IL-4-PE) (504104, BioLegend, San Diego, CA, USA), anti-IL-5-PE conjugate (eBioscience, 12-7052-82), anti-IL-13-PE conjugate (eBioscience, 12-7133-82), or anti-interferon (IFN)-γ-PE (BioLegend, 505807) for 30 min at 4°C. The stained Th1/Th2 cells were washed with phosphate buffered saline (PBS), and cellular fluorescence was determined by using fluorescence-activated cell sorter (BD FACSVantage SE). FACS data were acquired and analyzed by CellQuest^™^ software (BD Bioscience).

### RNA isolation and real-time PCR analysis

Activated CD4 T cells were transduced with Oct1-MIEG3 or control MIEG3 retroviral vector. GFP+ cells were sorted and re-stimulated for 4 h with anti-CD3 antibody for quantitative real-time PCR. The cells were lysed with TRIzol reagent (1 ml) (Invitrogen) and chloroform (200 μl), followed by centrifugation at 12,000 × g for 15 min at 4°C. Isopropanol (500 μl) was added to the aqueous phase, and the resultant total RNA was centrifuged at 12,000 × g for 10 min at 4°C. The RNA pellet was washed with 70% ethanol and centrifuged at 7,500 × g for 5 min at 4°C.

Reverse transcription was performed with the TOPscript^™^ Reverse Transcriptase Kit (#RT0025, Enzynomics, Jerusalem, Israel). Total RNA and 100 μM Oligo(dT)_18_ were heated for 5 min at 70°C. Next, TOPscript^™^ RT buffer (final concentration: 50 mM Tris-HCl, pH 7.5, 3 mM MgCl_2_, 10 mM DTT, and 75 mM KCl), dNTP mixture (2 mM each), RNase inhibitor (20 units), and TOPscript^™^ reverse transcriptase (200 units) were added, and the mixture was incubated at 50°C for 60 min and 95°C for 5 min.

Quantitative PCR reactions were performed by using a 7500 Real-Time PCR System (Applied Biosystems, Foster City, CA, USA), a HiFast Probe Lo-Rox Kit (#Q200220, Genepole, Korea), and the primers (400 nM each) listed in S3 Table. PCR reactions were performed at 1) 95°C for 2 min for polymerase activation, 2) 95°C for 10 sec for denaturation, and 3) 60°C for 34 sec for annealing/extension.

### Generation of Oct-1 binding site(s) mutant constructs

The mutation and deletion of Oct-1 binding sites of RHS5 and *Il4* promoter were made in the RHS5-IL4P construct using QuikChange Site-Directed Mutagenesis Kit (Agilent Technologies, Inc.). Deletion of Oct-1 binding site at the *Il4* promoter was generated by PCR using following primers. Forward: 5’-GGA-AAT-TAC-ACC-ATA-ATC-GGC-CTT-TCA-GAC-TAA-TC-3’; Reverse: 5’-GGA-AAA-TTT-ACC-TGT-TTC-TCT-TTT-TTC-TCC-TGG-AAG-3’. Mutation of the Oct-1 binding site at the RHS5 was generated by PCR using following primers. Forward: 5’-GTG-CAT-TTT-CGA-GAA-GCG-CTG-GCT-AGC-ATC-TGT-CAT-TAG-3’; Reverse: 5’-CTA-ATG-ACA-GAT-GCT-AGC-CAG-CGC-TTC-TCG-AAA-ATG-CAC-3’.

### Transient reporter assay

The transient reporter assay was performed as described previously [11]. The expression vector was constructed from a cytomegalovirus (CMV)-based expression vector (pCMV-SPORT6). Expression vectors (Oct1-CMV, Oct2-CMV, GATA-3-CMV, or control CMV), reporter plasmids (RHS5-IL-4 reporter construct), and *Renilla* luciferase reporter plasmid were transfected into the EL4 mouse thymoma cell line (10 × 10^6^ EL4 cells per transfection). Cells were transfected via electroporation by using a Bio-Rad Gene Pulser (Bio-Rad Laboratories, Inc., Berkeley, CA, USA) set at 950 μF and 270 V. Transfected cells were allowed to recover overnight in a complete medium for 18 h, followed by stimulation with 50 ng/ml PMA and 1 μM ionomycin for 4 h. Cells were then harvested and washed with PBS. Next, a luciferase reporter assay was performed by using the Dual-Luciferase Reporter Assay System (Promega, Madison, WI, USA) according to the manufacturer’s instructions. Transfection efficiency was normalized by dividing *Firefly* luciferase activity by *Renilla* luciferase activity.

### Small interfering RNA-mediated knockdown

Thirty microlitres of small interfering RNA (siRNA; stock concentration 100 μM) (Integrated DNA Technologies) were transfected into the EL4 mouse thymoma cell line (1 × 10^7^ EL4 cells per transfection). Cells were transfected via electroporation by using a Bio-Rad Gene Pulser (Bio-Rad Laboratories, Inc., Berkeley, CA, USA) set at 950 μF and 250 V. Transfected cells were allowed to recover overnight in a complete medium for 24 h. Then, the cells were stimulated with 50 ng/ml PMA and 1 μM ionomycin for 4 h, and total mRNA was isolated. The Oct siRNA is a mixture of two kinds of double-stranded RNA. The sequences of siRNA are listed in S4 Table.

### Statistical analysis

Data are expressed as the means ± the standard deviation (SD). Differences between groups were determined by Student's *t*-test.

## Results

### Oct-1, GATA-3, and Ets-1 bind to RHS5

**Fig 1:**
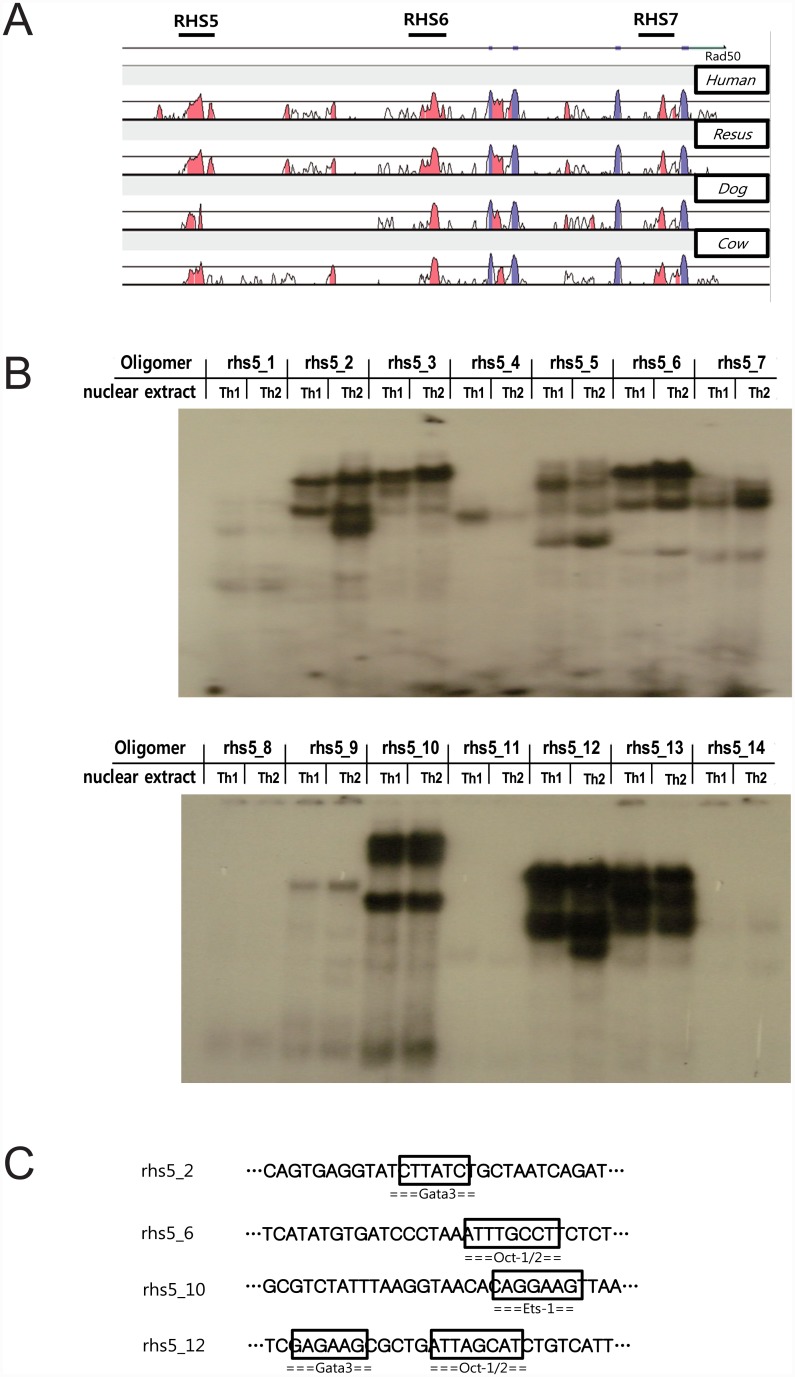
Screening for transcription factors that bind to RHS5. (A) DNA sequence alignment between mouse and other vertebrate species (human, rhesus, dog, and cow) in the Th2 LCR reveals several conserved regions, shown in blue and red (blue: exon, red: intergenic region). (B) RHS5 binding factors were screened by EMSA. Oligomers spanning the highly homologous region in the RHS5 (oligomers rhs5_1–rhs5_14) were labeled with ^32^P and incubated with nuclear extracts (2 μg) obtained from *in vitro*-differentiated Th1 or Th2 cells. The nuclear extracts were then subjected to EMSA. (C) Alibaba2.1 software was used to reveal transcription factor-binding sites within each oligomer, and to predict protein-oligomer complex formation for oligomers rhs5_2, rhs5_6, rhs5_10, and rhs5_12.

Fig 1A shows the locations of the DHSs in the Th2 LCR and their sequence homology between mouse and various vertebrate species. One of these sites, RHS5, is evolutionarily conserved among the species, suggesting that it may have an important role in Th2 cell function. To explore which transcription factors bind to RHS5, we performed EMSA by incubating nuclear extracts from Th1 and Th2 cells with radiolabeled synthetic oligomers spanning 290 base pairs of the highly homologous region in RHS5 (oligomers rhs5_1–rhs5_14, each oligomer overlapping ten nucleotides with the next). Consequently, several oligomer-bound complexes were identified on RHS5, some of which were either specific for Th2 cells (i.e., rhs5_2 and rhs5_12) or enriched in Th2 cells (rhs5_6 and rhs5_10) (Fig 1B).

**Fig 2:**
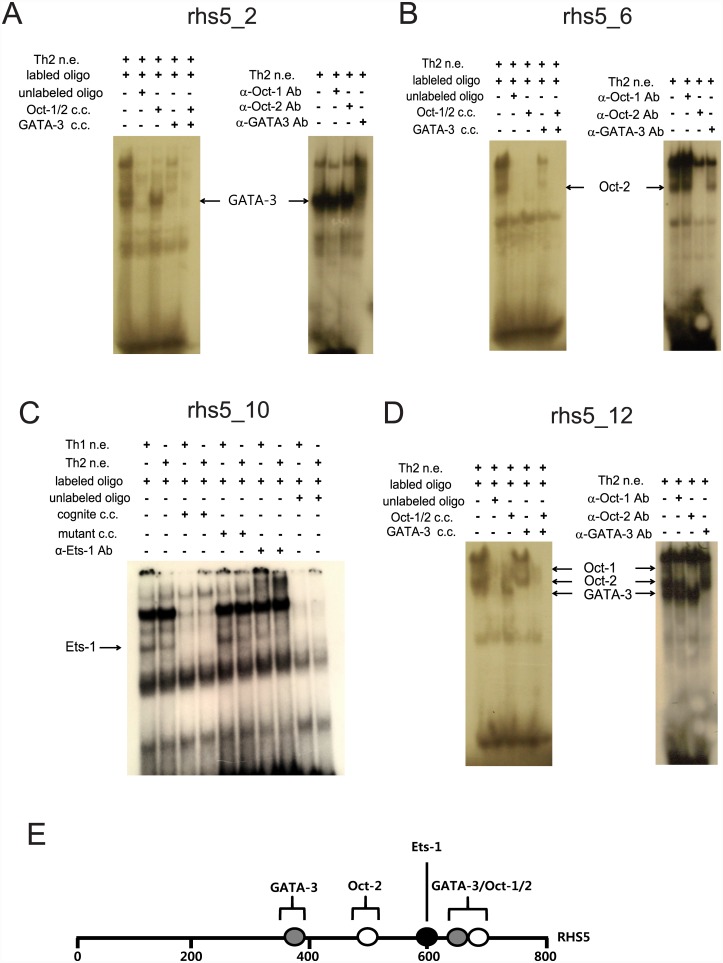
GATA-3, Ets-1, and Oct1/2 bind within RHS5. (A–D) Oligomers (rhs5_2, rhs5_6, rhs5_10, and rhs5_12) were labeled with ^32^P and incubated with nuclear extracts (2 μg) from Th1 or Th2 cells, and EMSA was performed. Each binding complex was identified by an oligonucleotide competition assay and an antibody-supershift assay. Arrows indicate bands containing specific transcription factors. n.e.: nuclear extract, c.c.: consensus competitor, Ab: antibody. (E) Schematic representation of transcription factor-binding sites within RHS5.

We employed Alibaba2.1 software (http://www.gene-regulation.com/pub/programs.html#alibaba2) to search for transcription factor binding sites and uncovered GATA-3-binding sites within rhs5_2 and rhs5_12, Oct-1/2-binding sites within rhs5_6 and rhs5_12, and an Ets-1-binding site within rhs5_10 (Fig 1C). Next, we performed an oligonucleotide competition assay with unlabeled competitor oligomers containing binding sites for the transcription factors and an antibody-supershift assay with specific antibodies directed against the transcription factors (Fig 2A–2D). The competition assay showed that GATA-3 bound to the Th2-specific sequences on the rhs5_2 and rhs5_12 oligomers, as expected (Fig 2A and 2D), while Oct-1 and Oct-2 bound adjacent to the GATA-3-binding site on the rhs5_12 oligomer (Fig 2D). Meanwhile, the antibody-supershift assay revealed that Oct-2, but not Oct-1, bound to the rhs5_6 oligomer (Fig 2B), and that Ets-1 bound to the rhs5_10 oligomer (Fig 2C). Taken together, these results suggest that RHS5 can associate with several transcription factors, including GATA-3, Ets-1, Oct-1, and Oct-2 (summarized in Fig 2E).

### Oct-1 binds to the *Il4* promoter and Th2 LCR

GATA-3 and Ets-1 reportedly participate in Th2 cell differentiation. GATA-3 binds to the GATAAG sequence found in several regulatory elements in Th2 cells, and thereby directs Th2 cell fate as a master transcription factor [9,12–14]. Ets-1 is also important for Th2 cell differentiation [15] via association with NC(A/C)GGA(A/T)G(C/T)N sequences, where GGA forms the core sequence of the Ets-1 binding site [16]. However, the function of Oct-1/2 in Th2 differentiation is poorly understood.

**Fig 3:**
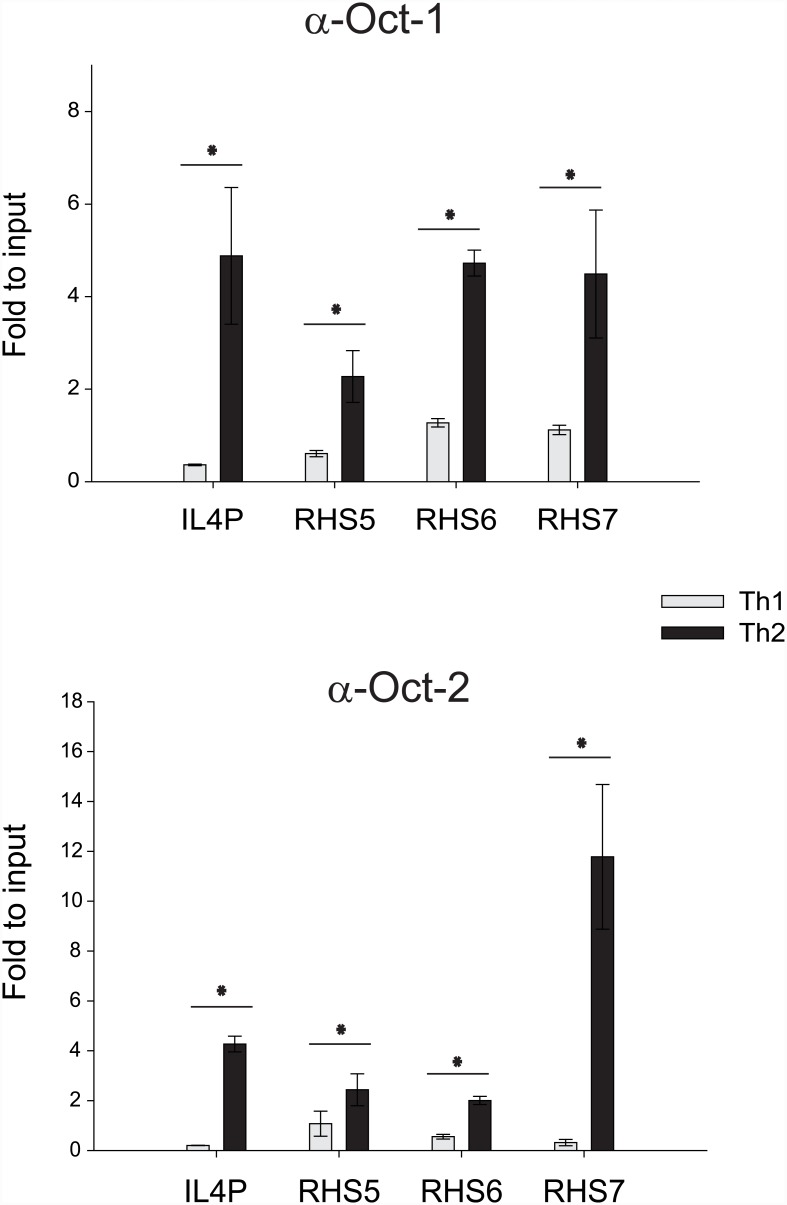
Oct-1/2 bind to the *Il4* promoter and DHSs within the Th2 LCR. Binding of Oct-1/2 to the *Il4* promoter (IL4P) and RHS5, RHS6, and RHS7 within the Th2 LCR was examined by a chromatin immunoprecipitation assay in Th1 and Th2 cells. Data are representative of three independent experiments with similar results. Statistical differences between groups were analyzed by Student’s *t*-test (*: P < 0.05).

We previously demonstrated that GATA-3 binds to the Th2 cytokine locus globally in a Th2-specific manner [9]. In addition, Kim et al. recently demonstrated that Oct-1 binds to octamer motifs within the RHS6 sequence [17]. To investigate whether Oct-1 contributes to the regulation of the Th2 cytokine locus, we utilized a chromatin immunoprecipitation assay to assess the association between Oct-1 and several regulatory regions within the Th2 cytokine locus. Splenic naïve CD4 T cells were isolated from C57BL/6 mice and stimulated *in vitro* under either Th1- or Th2-polarizing conditions. The cells were cross-linked, and the DNA was fragmented. Chromatin was then immunoprecipitated with an anti-Oct-1 antibody, anti-Oct-2 antibody, or a control IgG. After reverse cross-linking, DNA was eluted, and the amount of DNA covering the *Il4* promoter, RHS5, RHS6, and RHS7 was quantified by quantitative PCR. Interestingly, Oct-1 and Oct-2 both bound to the *Il4* promoter and RHS5, RHS6, and RHS7 in a Th2-specific manner (Fig 3).

### Oct-1 induces Th2 cell differentiation

**Fig 4:**
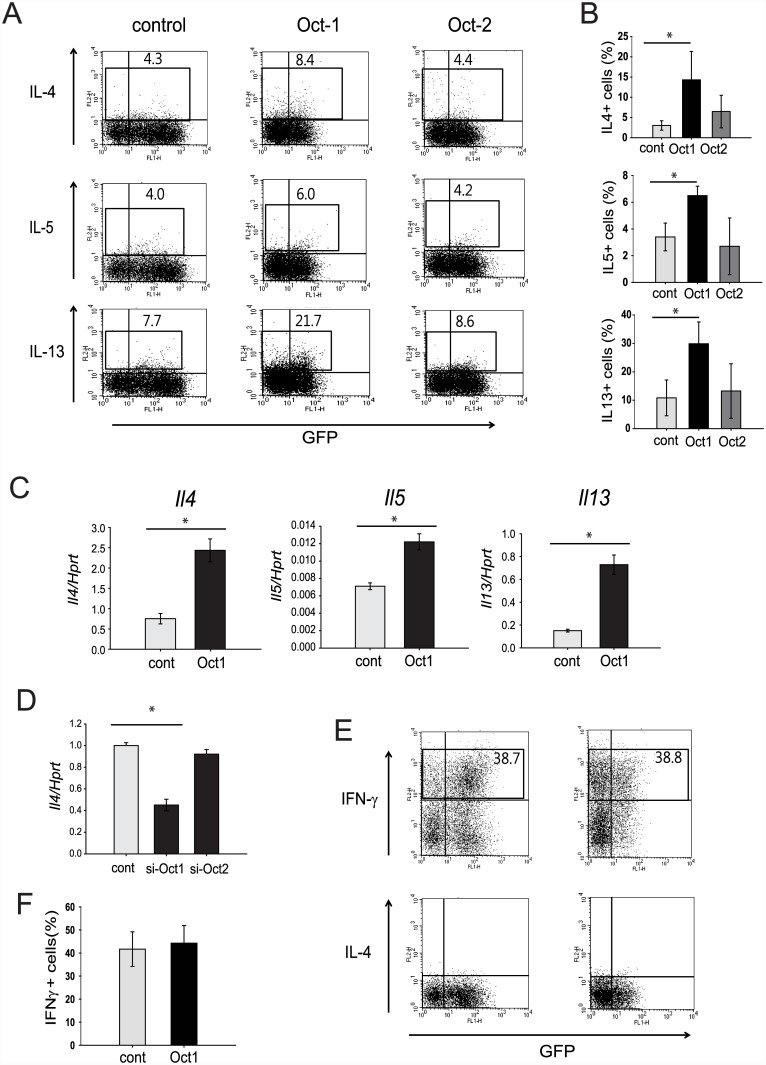
Oct-1 induces Th2 cytokine expression. (A) Naïve CD4 T cells were activated, transduced with Oct1-MIEG3, Oct2-MIEG3, or control (empty) MIEG3 retroviral vector, and differentiated into Th2 cells. Cytokine expression was measured by intracellular cytokine staining. The plots show the percentages of differentiated cells expressing a particular cytokine. Data are representative of three independent experiments with similar results. (B) The results in (A) were shown as bar graphs. Statistical differences between groups were analyzed by Student’s *t*-test (*: P < 0.05). (C) Expression levels of Th2 cytokine mRNA. Activated T cells were transduced with Oct-1-MIEG3 or control MIEG3 vector as in (A). GFP+ cells were sorted from samples used in (A) and re-stimulated with anti-CD3 antibody for 24 h. The mRNA expression levels for selected Th2 cytokines were measured by quantitative PCR. Statistical differences between groups were analyzed by Student’s *t*-test (*: P < 0.05). (D) The effect of *Oct1* siRNA on the expression of the *Il4* gene. EL4 cells were transfected with control or *Oct1* siRNA. The mRNA expression levels for the *Il4* gene was measured by quantitative RT-PCR. Bars indicate mean ± SD (n = 3). Statistical difference between groups was analyzed by Student’s *t*-test (*: P < 0.05). (E) Effect of Oct-1 overexpression on cytokine expression in Th1 cells. Naïve CD4 T cells were transduced with Oct1-MIEG3 or control MIEG3 retroviral vector as in (A) and differentiated into Th1 cells. Cytokine expression was measured by intracellular cytokine staining. The plots show the percentages of cells expressing a particular cytokine. Data are representative of three independent experiments with similar results. (F) IFN-γ-positive cells in (D) were shown as a bar graph.

We next explored whether Oct-1 and Oct-2 affect Th2 cell differentiation, as assessed by Th2 cytokine production. Naïve CD4 T cells were first transduced with a retroviral vector that expresses either Oct-1 or Oct-2, and then stimulated with anti-CD3 and anti-CD28 under Th2-polarizing conditions. Overexpression of Oct-1 increased the frequency of IL-4-, IL-5-, and IL-13-expressing cells (Fig 4A and 4B), as well as the transcript levels of the corresponding genes (Fig 4C). In addition, treatment with *Oct1* siRNA led to a significant reduction of *Il4* mRNA level in EL4 cells (Fig 4D), consistent with the overexpression experiments. Although Oct-2 also bound to the *Il4* promoter and the Th2 LCR (Fig 3), overexpression of this transcription factor did not affect the frequency of Th2 cytokine-expressing cells or the mRNA levels of Th2 cytokine genes (Fig 4A–4C).

Because previous studies showed that GATA-3 suppresses the Th1 differentiation program [18,19], we evaluated whether Oct-1 has the same ability. Nevertheless, Oct-1 upregulation failed to alter the frequency of IFN-γ-expressing cells in Th1-polarized CD4 T cells (Fig 4E and 4F). Collectively, the data of Fig 4 indicate that Oct-1, but not Oct-2, directly regulates Th2 cell differentiation in a Th2-specific manner, but that Oct-1, unlike GATA-3, does not share the capacity to directly influence the Th1 differentiation program.

### Oct-1 and GATA-3 synergistically transactivate the *Il4* promoter through RHS5

Our previous data demonstrated that GATA-3 regulates Th2 cell differentiation by enhancing transactivational activity of the *Il4* promoter through RHS7 [9]. Therefore, we next examined whether Oct-1/2 act on RHS5 to increase transcriptional activity of the *Il4* promoter by using a transient transfection assay. Oct-1 or Oct-2 expression vectors were transfected into the EL4 T cell lymphoma cell line together with an RHS5-IL4P reporter construct in which RHS5 is linked to the *Il4* promoter-luciferase gene. The EL4 cells were subsequently stimulated with PMA and ionomycin.

**Fig 5:**
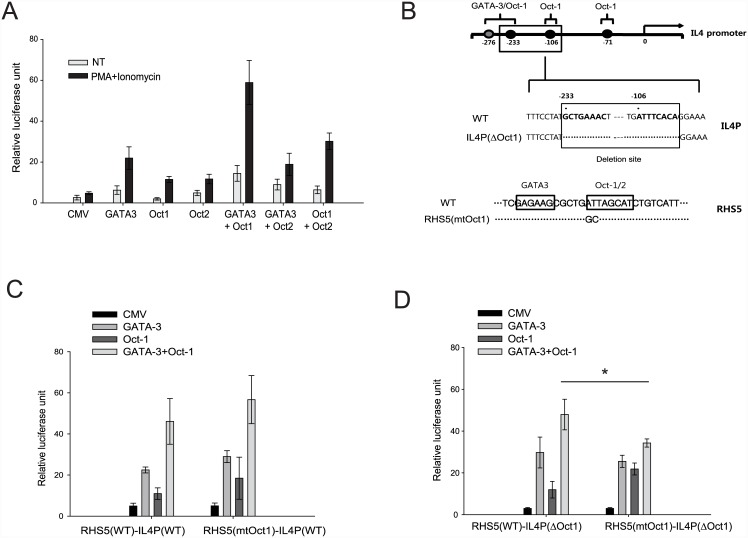
Oct-1 stimulates transactivation activity of the *Il4* promoter through RHS5. (A) EL4 cells were transfected with expression vectors (Oct1-CMV, Oct2-CMV, GATA-3-CMV, or control CMV) and the RHS5-IL4 reporter construct by electroporation. Cells were allowed to rest for 18 h, followed by treatment with PMA (50 ng/ml) and ionomycin (1 μM) for 4 h. Luciferase activity was then measured. Data are representative of three independent experiments with similar results (NT = no treatment). (B) Mutation sites of Oct-1-binding sites within *Il4* promoter and RHS5. Oct-1-binding sites, close to GATA-3-binding site, within *Il4* promoter were deleted as indicated (upper), or Oct-1 binding site adjacent to GATA3 binding site (Fig 2E) within RHS5 was mutated as indicated (lower). (C) RHS5(WT)-IL4P(WT) reporter construct or RHS5(mtOct1)-IL4P(WT) construct (RHS5-IL4P reporter construct bearing mutation at Oct-1 binding site within RHS5) were transfected into EL4 cells with expression vectors (Oct1-CMV, GATA-3-CMV, or control CMV) by electroporation. Cells were allowed to rest for 18 h, followed by treatment with PMA (50 ng/ml) and ionomycin (1μM) for 4 h. Luciferase activity was then measured. (D) RHS5(WT)-IL4P(ΔOct1) (RHS5-IL4P construct in which Oct1 binding sites were deleted within the *Il4* promoter) or RHS5(mtOct1)-IL4P(ΔOct1) (RHS5-IL4 reporter construct bearing both mutation at Oct-1 binding site within RHS5 and deletion of Oct-1 binding sites within the *Il4* promoter) were transfected into EL4 cells with expression vectors (Oct1-CMV, GATA-3-CMV, or control CMV) by electroporation. Luciferase activity was measured as (C). Data are representative of three independent experiments with similar results (NT = no treatment). Bars are shown to indicate mean ± SD (n = 3). Statistical difference between groups was analyzed by Student’s *t*-test (*: P < 0.05).

Introduction of Oct-1 or Oct-2 increased the transactivational activity of the *Il4* promoter to a similar extent as GATA-3 (Fig 5A). Our EMSA data revealing that Oct-1/2 and GATA-3 bind in close proximity within the RHS5 sequence (Fig 2) prompted us to next examine whether these transcription factors synergistically function in *Il4* gene expression. Vectors overexpressing Oct-1/2 and GATA-3 were transfected into EL4 cells in various combinations (Oct-1 and GATA-3, Oct-1 and Oct-2, or Oct-2 and GATA-3). Co-expression of Oct-1 and GATA-3 increased transactivation activity more effectively than either factor alone. Furthermore, co-expression of Oct-1/2, but not Oct-2/GATA-3, was also more effective than Oct-1 alone. These findings imply that Oct-1 impacts the transcriptional activity of the *Il4* promoter through RHS5, and acts in synergy with GATA-3 or Oct-2.

To examine the effect of Oct-1 binding in the RHS5 or in the IL4P, we mutated Oct-1 binding sites within RHS5 and/or *il4* promoter of the RHS5-IL4P construct (Fig 5B) and performed transient reporter assay. Although, mutation of Oct-1 binding site at RHS5 only had no effect on synergistic activation of *Il4* promoter activity by Oct-1 and GATA-3 (Fig 5C), mutation/deletion of Oct-1 binding sites at both RHS5 and *Il4* promoter reduced synergistic activation of *Il4* promoter activity by Oct-1 and GATA-3 substantially (Fig 5D). These results suggest that Oct-1 binding to both RHS5 and *Il4* promoter is required for proper cooperation between Oct-1 and GATA-3 for stimulation of *Il4* promoter activity and that the relative contribution of Oct-1 in this cooperation may be varied depending on their binding sites in the DNA.

## Discussion

This study set out to identify transcription factors with binding affinity toward RHS5, and to examine the capacity of these transcription factors to influence Th2 cell differentiation. We used a database analysis, an EMSA, an oligonucleotide competition assay, and an antibody-supershift assay to show that GATA-3, Ets-1, Oct-1, and Oct-2 bind to RHS5. Moreover, Oct-1 upregulation increased the gene expression of Th2 cytokines, as well as the frequency of IL-4-, IL-5-, and IL-13-expressing cells. In addition, Oct-1 bound to the *Il4* promoter and the Th2 LCR, and synergistically transactivated the *Il4* promoter through RHS5 in combination with GATA-3 or Oct-2. These results strongly suggest that Oct-1 is a crucial transcription factor for the differentiation of Th2 cells.

Our previous work demonstrated that the Th2 LCR is essential for the control of Th2 cytokine expression, chromatin remodeling, and intrachromosomal interactions within the Th2 cytokine locus [4,6,7]. The Th2 LCR itself is regulated by binding to tissue-specific transcription factors, including GATA-3, signal transducer and activator of transcription (STAT)-6, nuclear factor of activated T cells (NFAT), and YY1 [10,20–22]. Our current study adds to this knowledge by disclosing the complex interplay of transcription factors that bind to and modulate the Th2 LCR.

Using EMSA, we found that the GATA-3, Ets-1, and Oct-1/2 bind closely together within the RHS5 site. GATA-3 is a well-known lineage-determining transcription factor that drives Th2 cell differentiation [14], and Th2 cytokine expression is dramatically reduced in conditional GATA-3 knockout mice [23,24]. We previously showed that GATA-3 regulates Th2 cell differentiation by binding to several regulatory regions of the Th2 cytokine locus including the Th2 LCR [9]. Ets-1, an ETS family member, is imperative for T cell proliferation and survival [25,26]. Inactivation of Ets-1 induces T cell apoptosis and B cell differentiation [27], while the Ets-1 KO mouse has functional defects in CD4 T cells [15,28–30]. In the absence of Ets-1, Th1 cells produce low levels of IFN-γ, IL-2, and tumor necrosis factor-α [28], and Th2 cells produce abnormally low levels of Th2 cytokines (i.e., IL-4, IL-5, and IL-13) [15].

Unlike GATA-3 and Ets-1, the effect of the Oct proteins on Th2 cell differentiation remains unclear. Nevertheless, earlier work indicated that the Oct proteins may contribute to the regulation of Th2 cytokine genes. Oct-1 and Oct-2 belong to the POU domain family of proteins; Oct-1 is expressed ubiquitously, whereas Oct-2 is preferentially expressed in B lymphocytes [31]. Oct-1/2 positively or negatively modulate promoter activities of lymphocyte-specific and other genes via binding to the octamer element, ATGCAAAT. Oct-1 and Oct-2 form a complex on regulatory elements within the *Il5* locus, and positively regulate *Il5* expression in both mouse and human [32,33]. These octamer factors also bind to and stimulate *Il2* promoter activity through cooperative binding with activator protein-1 [34]. In the D10 mouse Th2 cell line, Oct-1 and Oct-2 influence the formation of Th2-specific protein-DNA complexes and mediate promoter activity of the proximal *Il4* promoter [35]. Besides the aforementioned positive regulation, Oct-1 also represses the expression of E-selectin and vascular cell adhesion molecule-1 by interacting with p65 [36]. In addition, Oct-1 binds with C/EBP to an element in the *Il8* promoter so as to strongly block the transcriptional activity of the latter [37].

Here, we showed that Oct-1 upregulation increased Th2 cytokine-expressing cell populations and the expression levels of these cytokines under Th2-polarizing conditions. Furthermore, Oct-1 bound to the *Il4* promoter and the Th2 LCR under Th2-polarizing conditions, and elevated the transactivation activity of the promoter either alone or in combination with GATA-3 or Oct-2. Although retroviral transduction of Oct-2 alone did not increase Th2 cytokine expression, it did so in combination with Oct-1 in a transient reporter assay. One possible explanation for these results is that Oct-2 may assist in the formation or stabilization of transcriptional complexes containing Oct-1 or GATA-3, which are then involved in the regulation of Th2 cytokines.

To examine the effects of Th2 differentiation by Oct-1 is due to differential expression of Oct-1 in Th2 cells, we examined expression of *Oct1* and *Oct2* in Th1 and Th2 cells. The expression levels were not much different between Th1 and Th2 cells, suggesting that expression level of Oct1/Oct2 may not be a critical factor in regulation of Th2 cytokine gene expression by Oct1/Oct2. Mutation analysis at the Oct-1 binding sites at the RHS5 and *Il4* promoter suggests that Oct-1 binding to both RHS5 and *Il4* promoter is required for cooperation between Oct-1 and GATA-3 for proper stimulation of *Il4* promoter activity and that the relative contribution of Oct-1 binding in this cooperation may be varied depending on their binding sites in the DNA. Transcription factors bound to LCR interact with other transcription factors bound to promoters, forming a complex(es) [21]. It is reasonable to expect that Oct-1 bound to Th2 LCR interact with GATA-3, YY-1, and Oct-1 that bound to the LCR and *Il4* promoter [10,21,34]. Therefore, relative contribution of Oct-1 binding to *Il4* transcription may be dependent on the role of Oct-1 in the complex which is dependent on the binding site of DNA. This complex formation and cooperative nature of transcription factors may also explain the role of Oct-1 in the absence of Th2-specific expression. In this case constitutively expressed Oct-1 may bind to the complex formed by Th2-specific transcription factors such as GATA-3 and YY-1 and enhances the function of the complex.

Usui et al. [19] previously showed that retroviral expression of GATA-3 under Th1-polarizing conditions suppressed Th1 cell development through downregulation of STAT4 [19]. Unlike GATA-3, retroviral expression of Oct-1 showed no effect against Th1 cytokine expression under Th1-polarizing conditions, whereas Brunner et al. [38] suggested that BOB.1/OBF.1 controls the balance of Th1- and Th2-mediated immunity through binding of Oct-1 to the *Ifng* and *Il2* promoters [38]. Therefore, although Oct-1 did not directly affect Th1 cytokine expression, it is still possible that the transcription factor may direct the expression of other genes to modulate the Th1-/Th2-mediated immunity balance.

In conclusion, the present study demonstrated that RHS5 confers specificity for the binding of Th2-regulatory factors (e.g., GATA-3, Ets-1, and Oct-1/2) to the Th2 LCR. In particular, we showed for the first time that Oct-1 participates in the control of Th2 cytokine protein expression. Our findings provide additional evidence for the control of Th2 cytokine synthesis by multifactor complexes on the Th2 LCR, and may assist in the development of new therapeutic strategies for the management of Th2 cell-related disorders.

## Supporting Information

S1 Table Oligomers used for EMSA. (DOCX)

S2 Table Primers for chromatin immunoprecipitation. (DOCX)

S3 Table Primers for quantitative RT-PCR. (DOCX)

S4 Table Sequences of siRNA. (DOCX)

S1 ARRIVE Checklist (DOC)

S1 FACS Data (ZIP)
