# Supplementary material for: Transcription Factors Oct-1 and GATA-3 Cooperatively Regulate Th2 Cytokine Gene Expression via the RHS5 within the Th2 Locus Control Region
Source: PLoS One. 2016 Feb 3;11(2):e0148576. doi: 10.1371/journal.pone.0148576 (PMC4740509; doi:10.1371/journal.pone.0148576)
Supplement: S1 Table — (DOCX) [file pone.0148576.s001.docx]

S1 Table. Oligomers used for EMSA.

| Oligomer name | Sequences | |
| --- | --- | --- |
| rhs5_1F | AGGAGCACTGCCACACTGTTCAGTGAGGTA | |
| rhs5_1R | TACCTCACTGAACAGTGTGGCAGTGCTCCT | |
| rhs5_2 F | CAGTGAGGTATCTTATCTGCTAATCAGATT | |
| rhs5_2 R | AATCTGATTAGCAGATAAGATACCTCACTG | |
| rhs5_3 F | TAATCAGATTCATTCTTCTAAATAAGGAGA | |
| rhs5_3 R | TCTCCTTATTTAGAAGAATGAATCTGATTA | |
| rhs5_4 F | GTCTGTGGGGTTTCTCCTGCAAACAGTGAT | |
| rhs5_4 R | ATCACTGTTTGCAGGAGAAACCCCACAGAC | |
| rhs5_5 F | AAACAGTGATGTCCTAAGATTCATATGTGA | |
| rhs5_5 R | TCACATATGAATCTTAGGACATCACTGTTT | |
| rhs5_6 F | TCATATGTGATCCCTAAATTTGCCTTCTCT | |
| rhs5_6 R | AGAGAAGGCAAATTTAGGGATCACATATGA | |
| rhs5_7 F | TGCCTTCTCTCCTTGCCAAAAAAAAAGGTC | |
| rhs5_7 R | GACCTTTTTTTTTGGCAAGGAGAGAAGGCA | |
| rhs5_8 F | CGTCTCAGAGTAAAGCACACTGTAGTGTCA | |
| rhs5_8 R | TGACACTACAGTGTGCTTTACTCTGAGACG | |
| rhs5_9 F | TGTAGTGTCACTGTTGCACAGCGTCTATTT | |
| rhs5_9 R | AAATAGACGCTGTGCAACAGTGACACTACA | |
| rhs5_10 F | GCGTCTATTTAAGGTAACACAGGAAGTTAA | |
| rhs5_10 R | TTAACTTCCTGTGTTACCTTAAATAGACGC | |
| rhs5_11 F | AGGAAGTTAACAGTGCATTTTCGAGAAGCG | |
| rhs5_11 R | CGCTTCTCGAAAATGCACTGTTAACTTCCT | |
| rhs5_12 F | TCGAGAAGCGCTGATTAGCATCTGTCATTA | |
| rhs5_12 R | TAATGACAGATGCTAATCAGCGCTTCTCGA | |
| rhs5_13 F | TCTGTCATTAGTAATCATCTAAAGCGCAGT | |
| rhs5_13 R | ACTGCGCTTTAGATGATTACTAATGACAGA | |
| rhs5_14 F | AAAGCGCAGTGAGCAGAAGCATTGCCAGAG | |
| rhs5_14 R | CTCTGGCAATGCTTCTGCTCACTGCGCTTT | |
| Oct consensus oligomer F | TGTCGAATGCAAATCACTAGA | |
| Oct consensus oligomer R | ACAGCTTACGTTTAGTGATCT | |
| Oct mutant oligomer F | TGTCGAATGCAAGCCACTAGA | |
| Oct mutant oligomer R | ACAGCTTACGTTCGGTGATCT | |
| Ets-1 consensus oligomer F | GATCTCGAGCAGGAAGTTCGA | |
| Ets-1 consensus oligomer R | CTAGAGCTCGTCCTTCAAGCT | |
| Ets-1 mutant oligomer F | GATCTCGAGCAAGAAGTTCGA | |
| Ets-1 mutant oligomer R | CTAGAGCTCGTTCTTCAAGCT | |
| Gata3 consensus oligomer F | CACTTGATAACAGAAAGTGATAACTCT | |
| Gata3 consensus oligomer R | AGAGTTATCACTTTCTGTTATCAAGTG |  |
| Gata3 mutant oligomer F | CACTTGATAACAGAAAGTCTTAACTCT |  |
| Gata3 mutant oligomer R | AGAGTTAAGACTTTCTGTTATCAAGTG |  |
