# Supplementary material for: Transcription Factors Oct-1 and GATA-3 Cooperatively Regulate Th2 Cytokine Gene Expression via the RHS5 within the Th2 Locus Control Region
Source: PLoS One. 2016 Feb 3;11(2):e0148576. doi: 10.1371/journal.pone.0148576 (PMC4740509; doi:10.1371/journal.pone.0148576)
Supplement: S2 Table — (DOCX) [file pone.0148576.s002.docx]

S2 Table. Primers for chromatin immunoprecipitation.

| Primer name | Sequences |
| --- | --- |
| IL-4 promoter Fw | GCAGGATGACAACTAGCTGGG |
| IL-4 promoter Rv | ACGGCACAGAGCTATTGATGG |
| RHS5 Fw | AATTTCCCTCCTTGTTTGTCGT |
| RHS5 Rv | CTGCTCACTGCGCTTTAGATG |
| RHS6 Fw | GGCTCCCTCTGCTCTGACT |
| RHS6a Rv | TAAAAAGGGAAAATGGTAACAAAA |
| RHS7 Fw | TGCCTGCCCTGCAAACA |
| RHS7 Rv | TGTGGGAGGAGATAGGACTCTTACC |
