# Supplementary material for: Transcription Factors Oct-1 and GATA-3 Cooperatively Regulate Th2 Cytokine Gene Expression via the RHS5 within the Th2 Locus Control Region
Source: PLoS One. 2016 Feb 3;11(2):e0148576. doi: 10.1371/journal.pone.0148576 (PMC4740509; doi:10.1371/journal.pone.0148576)
Supplement: S3 Table — (DOCX) [file pone.0148576.s003.docx]

S3 Table. Primers for quantitative RT-PCR.

| Primer name | Sequences |
| --- | --- |
| *il4* sense | AGATCATCGGCATTTTGAACG |
| *il4* anti-sense | TTTGGCACATCCATCTCCG |
| *il4* probe | FAM-5′-TCACAGGAGAAGGGACGCCATGC-3′-Tamra |
| *il5* sense | CGCTCACCGAGCTCTGTTG |
| *il5* anti-sense | CCAATGCATAGCTGGTGATTTTT |
| *il5* probe | FAM-5′-CAATGAGACGATGAGGCTTCCTGTCCC-3′-Tamra |
| *il13* sense | GCTTATTGAGGAGCTGAGCAACA |
| *il13* anti-sense | GGCCAGGTCCACACTCCATA |
| *il13* probe | FAM-5′-CAAGACCAGACTCCCCTGTGCAACG-3′-Tamra |
| *hprt* sense | CTGGTGAAAAGGACCTCTCG |
| *hprt* anti-sense | TGAAGTACTCATTATAGTCAAGGGCA |
| *hprt* probe | FAM-5′-TGTTGGATACAGGCCAGACTTTGTTGGAT-3′-Tamra |
