# Supplementary material for: Transcription Factors Oct-1 and GATA-3 Cooperatively Regulate Th2 Cytokine Gene Expression via the RHS5 within the Th2 Locus Control Region
Source: PLoS One. 2016 Feb 3;11(2):e0148576. doi: 10.1371/journal.pone.0148576 (PMC4740509; doi:10.1371/journal.pone.0148576)
Supplement: S4 Table — (DOCX) [file pone.0148576.s004.docx]

S4 Table. Sequences of siRNA.

| siRNA name | Sequences |
| --- | --- |
| Oct-1 (sense) | 5’-CCUGCAACCAGCACAGUUUAUCA(dTdC)-3’ |
| Oct-1 (anti-sense) | 5’-GAUGAUAAACUGUGCUGGUUGCAGGUU-3’ |
| Oct1-2 (sense) | 5’-CCUCCUACAGCCACAGCCAAGCA(dTdC)-3’ |
| Oct1-2 (anti-sense) | 5’-GAUGCUUGGCUGUGGCUGUAGGAGGUU-3’ |
| Oct2-1 (sense) | 5’-GGAGGAGCUGGAACAGUUUGCUC(dGdC)-3’ |
| Oct2-1 (anti-sense) | 5’-GCGAGCAAACUGUUCCAGCUCCUCCAG-3’ |
| Oct2-2 (sense) | 5’-GGACCAGCAUCGAGACGAAUGUC(dCdG)-3’ |
| Oct2-2 (anti-sense) | 5’-CGGACAUUCGUCUCGAUGCUGGUCCUC-3’ |
